# Supplementary material for: One-pot catalytic synthesis of urea derivatives from alkyl ammonium carbamates using low concentrations of CO2
Source: Commun Chem. 2021 May 14;4:66. doi: 10.1038/s42004-021-00505-2 (PMC9814107; doi:10.1038/s42004-021-00505-2)
Supplement: Supplementary file 1 — Supplementary Information [file 42004_2021_505_MOESM1_ESM.pdf]

Supplementary Information

One-pot catalytic synthesis of urea derivatives from alkyl ammonium carbamates using low concentrations of CO_2

Hiroki Koizumi[1], Katsuhiko Takeuchi[1]*, Kazuhiro Matsumoto[1], Norihisa Fukaya[1], Kazuhiko Sato[1], Masahito Uchida[2], Seiji Matsumoto[3], Satoshi Hamura[3], and Jun-Chul Choi[1]*

[1] National Institute of Advanced Industrial Science and Technology (AIST), Tsukuba Central 5, 1-1-1 Higashi, Tsukuba 305-8565, Ibaraki, Japan.

[2] Tosoh Corporation, Advanced Materials Research Laboratory, 2743-1 Hayakawa, Ayase, Kanagawa 252-1123, Japan.

[3] Tosoh Corporation, 3-8-2 Shiba, Minato-Ku, Tokyo 105-8623, Japan.

email: takeuchi-k@aist.go.jp; junchul.choi@aist.go.jp

Table of contents

Supplementary Methods **S2-S27**

General information S2

Synthesis of ammonium carbamate salts using high purity CO_2 gas S2

Synthesis of 2-ammonioethylcarbamate from CO_2/N_2 mixed gas (v:v = 15:85) or ambient air S6

Determination of conversion yield for ethylenediamine during CO_2 bubbling S10

Catalyst performance for ethylene urea synthesis S11

Investigation of activation energy for ethylene urea synthesis S12

Synthesis of urea derivatives S13

Copies of NMR spectra for urea derivatives S17

One-pot synthesis of urea derivatives S27

Pressure change during reaction of **3a** synthesis S27

Supplementary References **S28**

Supplementary Methods

General Information

All commercial amines and catalysts except $Cp_2Ti(OTf)_2$ were purchased from TCI, FUJIFILM Wako Pure Chemical or Sigma-Aldrich and used without any purification. $Cp_2Ti(OTf)_2$ were synthesized according to the literature method[1] and stored in a glove box. EtOH and *n*-hexane for ammonium carbamate salts synthesis were purchased from FUJIFILM Wako Pure Chemical as a special grade and used without further purifications. Solvents for urea synthesis except DMI, NMP, and 2-pyrroridone were purchased from FUJIFILM Wako Pure Chemical as super dehydrated grade and used in a glove box without any purifications. DMI, NMP and 2-pyrroridone were purchased from TCI, and used in a glove box after drying with activated molecular sieve 4A purchased from Kanto Chemical Industry. CO_2 (purity: 99.999) and CO_2/N_2 mixed gases (v:v = 50:50 and 15:85) were purchased SHOWA DENKO GAS PRODUCTS CO., LTD. and used without further purification. Simulated exhaust gas was purchased from TAKACHIHO CHEMICAL INDUSTRIAL CO., LTD. and contains 300 ppm of CO, 500 ppm of NO_2, 500 ppm of SO_2, 15 vol% of CO_2 and 85 vol% of N_2. ^{1}H and $^{13}C\{^{1}H\}$ NMR spectra were recorded in DMSO-d_6, methanol-d_4, or D_2O by Bruker AVANCE-III high-resolution spectrometer with 400 MHz magnetic field. Chemical shifts of compounds in NMR spectra were corrected by using reported residual solvent signals as reference[2]. Elemental analyses were carried out by PerkinElmer 2400II.

Synthesis of ammonium carbamate salts using a high purity CO_2 gas

All ammonium carbamate salts were synthesized according to a similar method. Thus, a synthetic procedure of 2-ammonioethylcarbamate is shown as a typical example herein. Different aspects such as substrate amount are described in sections for each compound. 3.00 g (50.0 mmol) of ethylenediamine was dissolved in 50 mL of EtOH at a 100 mL eggplant flask. This solution was bubbled by pure CO_2 gas for 10 min. A white precipitate was filtered and washed with EtOH. After drying under reduced pressure at room temperature for 15 h, 2-ammonioethylcarbamate (**1a**) was obtained as white powder.

2-Ammonioethylcarbamate (1a)

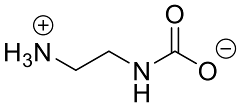

Yield: 4.82 g (4.63 mmol), 93%. Anal. Calcd. for $C_3H_8N_2O_2$: C, 34.61; H, 7.75; N, 26.91. Found: C,34.65; H, 7.95; N, 26.73.

Mixture of 2-ammoniopropylcarbamate and 2-ammonio-1-methylethylecarbamate (1b)

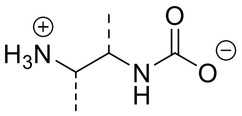

This mixture was treated in a glove box due to hygroscopicity except for CO_2 bubbling. After CO_2 bubbling for 50 mL of dehydrated *n*-hexane containing 2.96 g (40.0 mmol) of 1,2-diaminopropane, the vessel was tightly closed and transferred to a glove box. Filtrating to separate a precipitate, washing with *n*-hexane and drying under reduced pressure, the mixture of target compounds was obtained as impure white powder.
Yield: 3.74 g (79%: This yield was tentative isolated yield if **1b** was pure). Anal. Calcd. for $C_4H_{10}N_2O_2$: C, 40.67; H, 8.53; N, 23.71. Found: C, 37.64; H, 8.57; N, 23.08.

***Cis*-(2-ammoniocyclohexyl)carbamate (1c)**

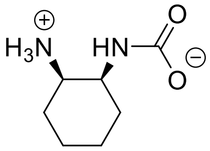

This compound was synthesized from 2.28 g (20.0 mmol) of *cis*-1,2-diaminocyclohexane in 50 mL of EtOH. After filtration, washing with EtOH and drying under reduced pressure, this compound was obtained as white powder.
Yield: 2.77 g (17.5 mmol), 88%. Anal. Calcd. for $C_7H_{14}N_2O_2$: C, 53.15; H, 8.92; N, 17.71. Found: C,52.91; H, 8.85; N, 17.55.

***Trans*-(2-ammoniocyclohexyl)carbamate (1d)**

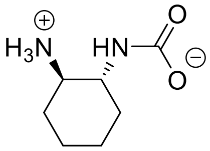

This compound was synthesized from 2.28 g (20.0 mmol) of *trans*-1,2-diaminocyclohexane in 50 mL of EtOH. After filtration, washing with EtOH and drying under reduced pressure, this compound was obtained as pale yellow powder.
Yield: 2.28 g (14.4 mmol), 72%. Anal. Calcd. for $C_7H_{14}N_2O_2$: C, 53.15; H, 8.92; N, 17.71. Found: C,53.01; H, 8.83; N, 17.50.

3-Ammoniopropylcarbamate (1e)

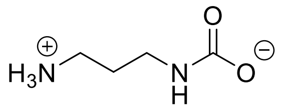

This compound was synthesized from 2.96 g (40.0 mmol) of 1,3-diaminopropane in 50 mL of EtOH. After filtration, washing with EtOH and drying under reduced pressure, this compound was obtained as white powder.

Yield: 4.47 g (37.8 mmol), 95%. Anal. Calcd. for $C_4H_{10}N_2O_2$: C, 40.67; H, 8.53; N, 23.71. Found: C,40.66; H, 9.02; N, 23.58.

***N,N'*-dimethyl-2-ammonioethylecarbamate (1f)**

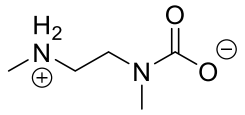

This compound was synthesized from 3.52 g (40.0 mmol) of *N,N'*-dimethylethylenediamine in 50 mL of *n*-hexane. After filtration, washing with *n*-hexane and drying under reduced pressure for 4 h, this compound was obtained as white powder.

Yield: 4.70 g (35.5 mmol), 89%. Anal. Calcd. for $C_5H_{12}N_2O_2$: C, 45.44; H, 9.15; N, 21.20. Found: C,45.37; H, 9.15; N, 21.07.

***N,N'*-dibenzyl-2-ammonioethylecarbamate (1g)**

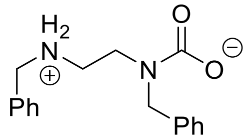

This compound was synthesized from 4.81 g (20.0 mmol) of *N,N'*-dibenzylethylenediamine in 50 mL of *n*-hexane. After filtration, washing with *n*-hexane and drying under reduced pressure for 4 h, this compound was obtained as white powder.

Yield: 5.49 g (19.3 mmol), 97%. Anal. Calcd. for $C_5H_{12}N_2O_2$: C, 71.81; H, 7.09; N, 9.85. Found: C,71.80; H, 7.20; N, 9.87.

Benzylammonio benzylcarbamate (2a)

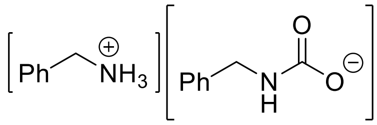

This compound was synthesized from 44.5 g (415 mmol) of benzylamine in 300 mL of *n*-hexane at an 500 mL eggplant flask for 10 min CO_2 bubbling. After filtration to afford a precipitate, CO_2 was bubbled to the filtrate again. These operations were conducted three times. Then, washing the obtained solid with *n*-hexane and drying under reduced pressure, this compound was obtained as white powder.

Yield: 49.6 g (19.2 mmol), 93%. Anal. Calcd. for $C_{15}H_{18}N_2O_2$: C, 69.74; H, 7.02; N, 10.84. Found: C, 69.38; H, 7.14; N, 10.82.

***n*-Hexylammonio *n*-hexylcarbamate (2b)**

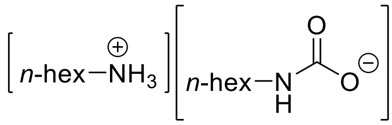

This compound was synthesized from 3.03 g (30.0 mmol) of *n*-hexylamine in 50 mL of *n*-hexane. After filtration, washing with *n*-hexane and drying under reduced pressure, this compound was obtained as white powder.

Yield: 3.40 g (13.8 mmol), 92%. Anal. Calcd. for $C_{13}H_{30}N_2O_2$: C, 63.37; H, 12.27; N, 11.37. Found: C,63.33; H, 12.39; N, 11.28

Cyclohexylammonio cyclohxylecarbamate (2c)

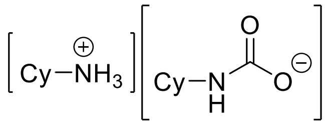

This compound was synthesized from 2.99 g (30.0 mmol) of *n*-hexylamine in 50 mL of *n*-hexane. After filtration, washing with *n*-hexane and drying under reduced pressure for 4 h, this compound was obtained as white powder.

Yield: 3.10 g (12.6 mmol), 84%. Anal. Calcd. $C_{13}H_{26}N_2O_2$: C, 64.43; H, 10.81; N, 11.56. Found: C,64.34; H, 11.18; N, 11.49.

2-Aminobenzylammonio 2-aminobenzylcarbamate (2d)

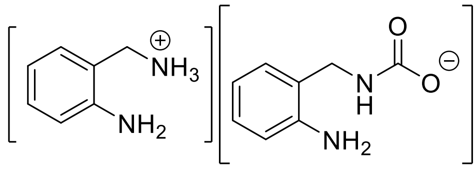

This compound was synthesized from 3.66 g (30.0 mmol) of 2-aminobenzylamine in 50 mL of EtOH. After filtration, washing with Et_2O and drying under reduced pressure, this compound was obtained as white powder.

Yield: 4.24 g (14.7 mmol), 98%. Anal. Calcd. for $C_{15}H_{20}N_4O_2$: C, 62.51; H, 6.98; N, 19.28. Found: C,62.48; H, 6.99; N, 19.43.

Synthesis of 2-ammonioethylcarbamate from CO_2/N_2 mixed gas (v:v = 15:85) or ambient air

Synthesis of 2-ammonioethylcarbamate (**1a**) from CO_2/N_2 mixed gas (v:v = 15:85) or simulated exhaust gas (CO_2: 15 vol%, N_2: 85 vol%, CO: 300 ppm, NO_2: 500 ppm, SO_2: 500 ppm) were conducted according to the similar method mentioned above except for bubbling time. Yield of **1a** were 96% (5.09 g, 48.9 mmol) or 92% (4.88 g, 46.8 mmol) using CO_2/N_2 mixed gas (v:v = 15:85) or simulated exhaust gas respectively, when 50 mL of EtOH containing 3.00 g (50.0 mmol) of ethylenediamine was bubbled for 30 min.

Synthesis of **1a** from ambient air were conducted in absence of solvent. 1.01 g (17 mmol) of ethylenediamine was in a 50 mL screw vial. Three of same ones were prepared and kept static in a draft chamber for about 1 week. Precipitated solids were filtered, washed with EtOH and dried under reduced pressure at room temperature for 15 h. 2-Ammonioethylcarbamate **1a** was afford as a white solid at 45% (2.39 g, 23.0 mmol) yield.

Supplementary Table 1| Elemental analysis of **1a** synthesized from various CO_2 concentration gas

Condition	Found[a]		
	C	H	N
Pure CO_2	34.65	7.62	26.68
CO_2/N_2 mixed gas (v:v = 15:85)	34.65	7.95	26.73
Simulated exhaust gas	34.44	8.04	26.73
Ambient air	34.68	7.97	26.44

a: Calcd. for $C_3H_8N_2O_2$: C, 34.61; H, 7.75; N, 26.91.

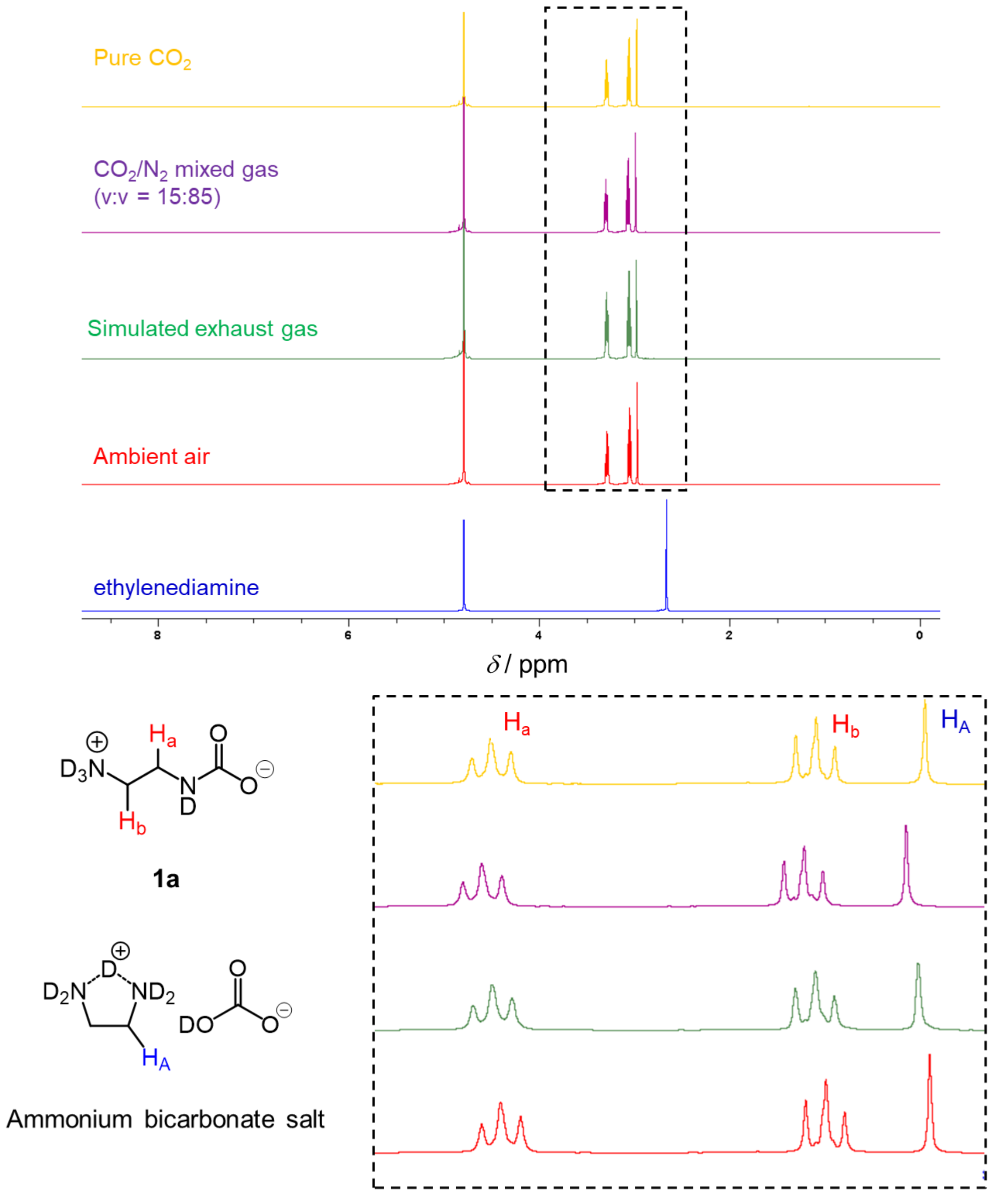

Supplementary Fig. 1| ^{1}H NMR spectra of **1a** synthesized from various CO_2 concentration gas and ethylenediamine measured in D_2O. These signals were assigned by ^{1}H-^{13}C HMQC spectrum (Supplementary Fig. 3).

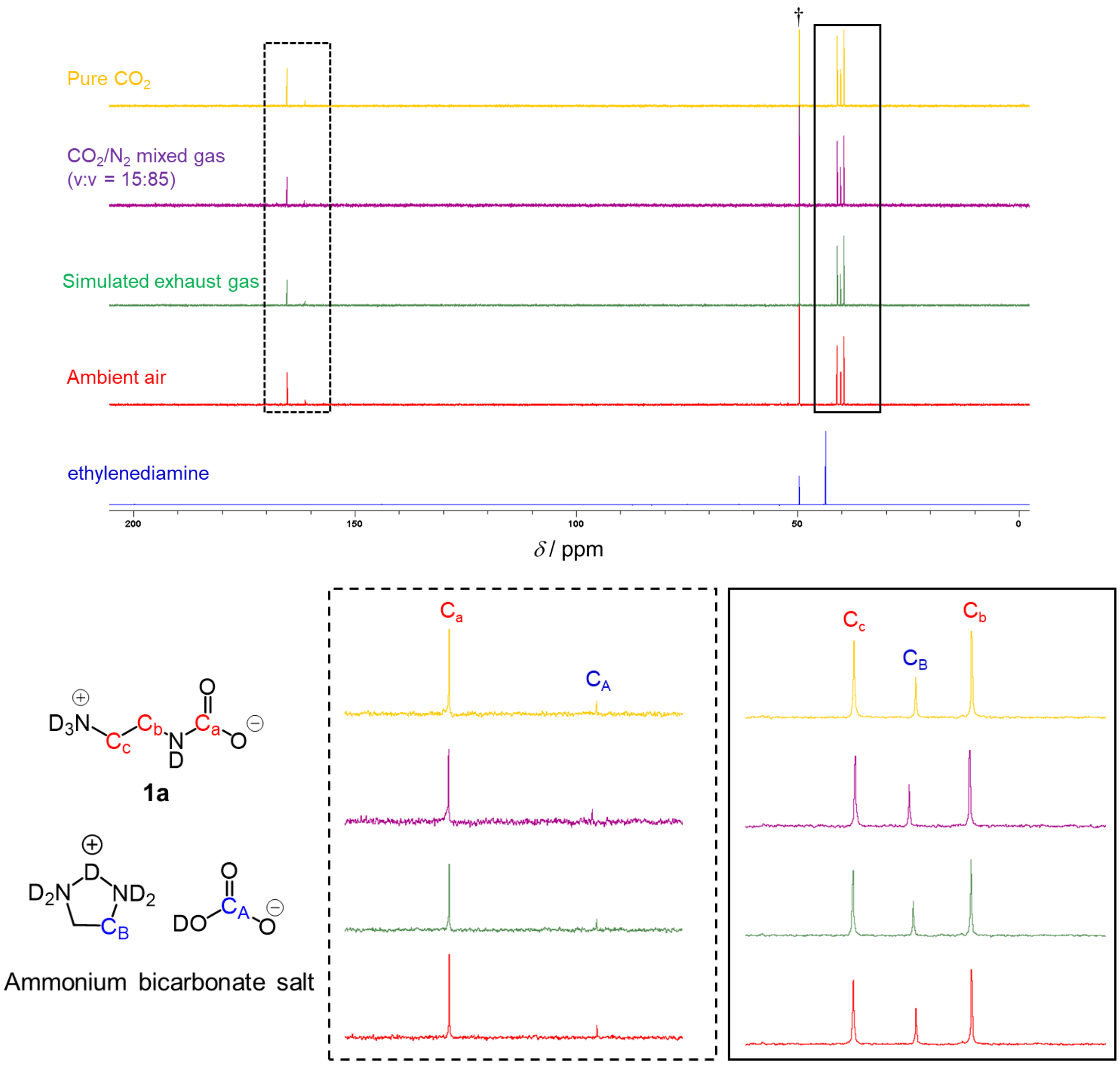

Supplementary Fig. 2| $^{13}C\{^{1}H\}$ NMR spectra of **1a** synthesized from various CO_2 concentration gas and ethylenediamine measured in D_2O. † at 49.5 ppm is MeOH signal as an internal standard. These signals were assigned by ^{1}H-^{13}C HMQC spectrum (Supplementary Fig. 3)

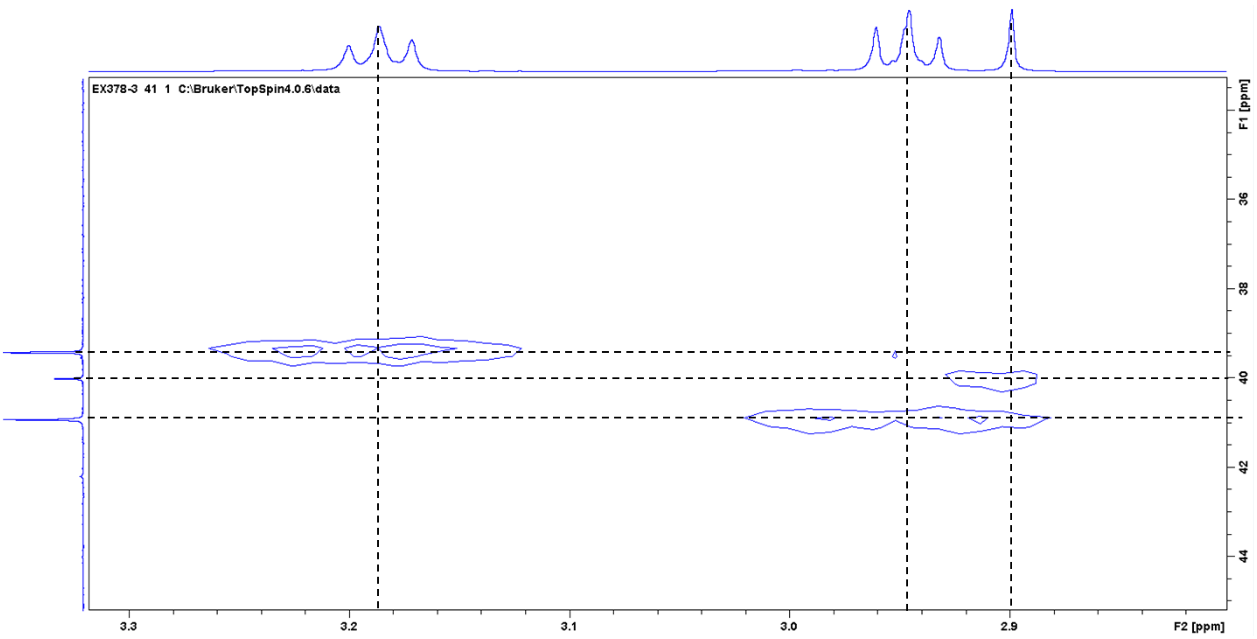

Supplementary Fig. 3| ^1H-^{13}C HMQC spectrum of **1a** measured in D_2O.

Determination of conversion rate for ethylenediamine during CO_2 bubbling

All reactions were conducted in 50 mL of EtOH containing 1.20 g (20.0 mmol) of ethylenediamine and 1,3,5-trimethylbenzene as an internal standard for ^{1}H NMR measurement. Various CO_2 concentration gases were bubbled to this EtOH solution at 50 mL/min of flow rate controlled by a mass-flow controller MC100SCCM-D/5M (ALICAT SCIEMTIFIC). A small amount of this solution was extracted at fixed intervals and then diluted by DMSO-d_6 to measure ^{1}H NMR. Conversion rate of ethylenediamine was determined by correction and comparing ratio between signals of ethylenediamine and 1,3,5-trimethylebenzene.

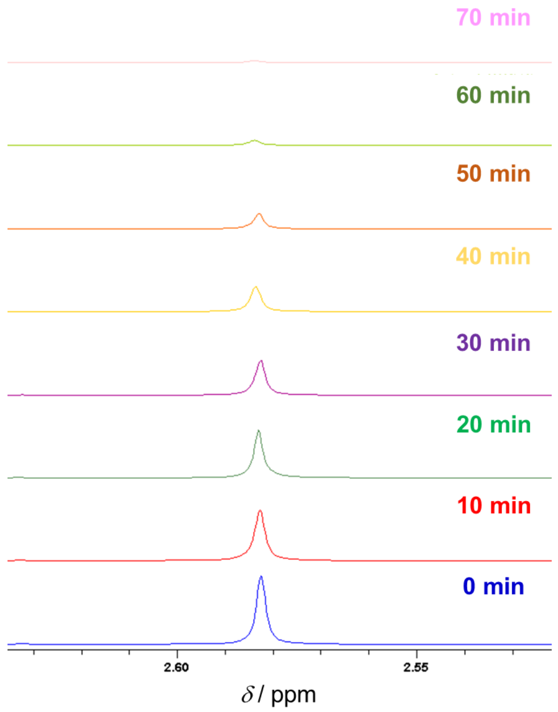

Supplementary Fig. 4| Intensity changes of ethylenediamine signal in NMR spectra measured during CO_2/N_2 mixed gas (v:v = 15:85) bubbling. All spectra were normalized at mesitylene signal and corrected for the horizontal axis to easily compare.

Catalyst performance for ethylene urea synthesis.

Catalytic activities of various catalysts for ethylene urea (**3a**) synthesis were investigated in 4.5 mL of DMI containing 208 mg (2.00 mmol) of **1a** at 170 ºC for 3 h by using 10mol% of a catalyst. Yield of **3a** was determined by ^{1}H NMR in DMSO-d_6 with 1,3,5-trimethylebenzene as an internal standard.

Supplementary Table 2| Catalyst performance for ethylene urea synthesis

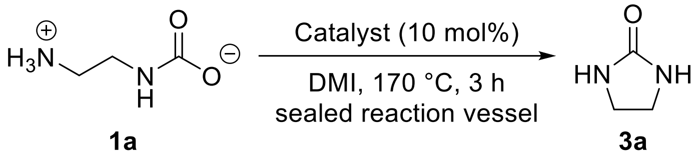

1a **3a**

Entry	Catalyst	Yield of **3a**
1	Bu_2SnO	46
2	$Bu_2Sn(C_{12}H_{23}O_2)_2$	24
3	$InCl_3$	3
4	Cp_2TiCl_2	42
5	$Cp_2Ti(OTf)_2$	52
6	$TiCl_4(THF)_2$	36
7	$TiCl_4(THF)_2$ + AgOTf (4 eq.)	46
8	$Ti(OEt)_4$	22
9	$Ti(O^tBu)_4$	22
10	Cp_2ZrCl_2	41
11	$Zr(OEt)_4$	30
12	$Hf(OTf)_4$	28
13	$Y(OTf)_3$	2
14	$Sc(OTf)_3$	2
15	$Fe(OTf)_3$	6
16	$Zn(OTf)_3$	1
17	$Ce(OTf)_3$	3
18	$Yb(OTf)_3$	2
19	$Ln(OTf)_3$	1
20	$Nd(OTf)_3$	1>
21	$Tm(OTf)_3$	2
22	K_2CO_3	27
23	Cs_2CO_3	34
24	CsOH	28
25	DBU	43
26	*N*,*N*-diisopropylethylamine	7
27	CeO_2 (25> nm, Sigma-Aldrich)	29

Investigation of activation energy for ethylene urea synthesis

Activation energy *E*a for ethylene urea synthesis were calculated from Arrhenius plot (Supplementary Fig. 3 and Eq. 1), which was based on reaction results using 95.8 mg (200 μmol, 10mol%) of $Cp_2Ti(OTf)_2$ as a catalyst, 208 mg (2.00 mmol) of **1a** and 4.5 mL of DMI as a solvent for 1 h at each temperature. Rate constants *k* were obtained by using the first order rate equation (Eq. 2) depending on the concentration of **1a** ($[M_{1a}]$), which was calculated by subtraction the concentration of **3a** from the initial concentration of **1a** ($[M_{1a0}]$). R = 8.314 J $K^{-1}mol^{-1}$. $[M_{1a0}]$ = 0.44 M.

$$ln\,k = -\frac{E_a}{RT} + lnA \qquad \text{Eq. 1}$$

$$ln[\mathrm{M_{1a}}] = -kt + ln\,[\mathrm{M_{1a0}}] \qquad \text{Eq. 2}$$

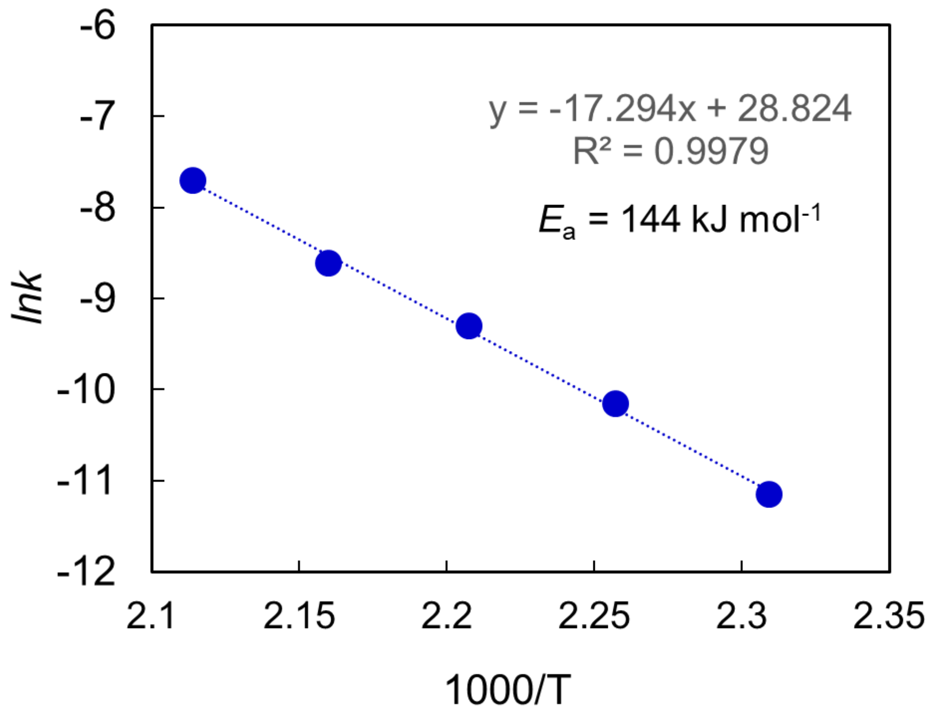

Supplementary Fig. 5| Arrhenius plot for ethylene urea synthesis.

Synthesis of urea derivatives

All urea derivatives were synthesized by a similar method. Therefore, a procedure of 2-imidazolidinone is shown as an example for typical urea derivative synthesis herein. The different aspects such as substrate amount are described in sections for each compound. 1.04 g (10.0 mmol) of **1a**, 95.2 mg (200 μmol, 2 mol%) of $Cp_2Ti(OTf)_2$ and 4.5 mL of DMI were added to an 5 mL autoclave reactor vessel and completely shielded with a stainless plate gasket. The vessel was dunked in a heated oil bath, and the reaction solution was stirred at 170 ºC for 24 h. After reaction, the vessel was removed from the oil bath and cooled to room temperature. The reaction mixture was extracted with MeOH (ca. 20 mL) and 1,3,5-trimethylebenzene (~100 mg) was added as an internal standard for ^{1}H NMR to determine NMR yield. After measuring the ^{1}H NMR spectrum, MeOH was removed under reduced pressure by an evaporator apparatus. Then, components other than the target compound were roughly eliminated using a Kugelrohr apparatus. The obtained solid was washed with Et_2O/*n*-hexane and dried under reduced pressure at 50 ºC to afford 2-imidazolidinone (**3a**) as a white solid.

2-Imidazolidinone (3a)[3]

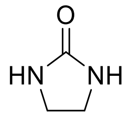

Yield: 703 mg (8.17 mmol), 82%. ^{1}H NMR (DMSO-d_6, 400 MHz): δ (ppm) 6.10 (br, s, 2H), 3.26 (s, 4H). ^{13}C{^{1}H} NMR (MeOH-d_4, 101 MHz): δ (ppm) 163.7, 48.0, 47.9, 21.7.

4-Methyle-2-imidazolidinone (3b)[4]

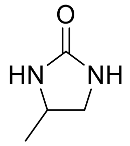

This compound was synthesized, using 1.18 g of crude **1b**, 95.8 mg (200 μmol, 2 mol%) of $Cp_2Ti(OTf)_2$ and 4.5 mL of DMI and obtained as a white solid according to the typical method.

Yield: 803 mg, 80% (This yield was tentative isolated yield if pure **1b** was used). ^{1}H NMR (DMSO-d_6, 400 MHz): δ (ppm) 6.26 (br, s, 1H), 6.06 (br, s, 1H), 3.71-3.63 (m, 1H), 3.38 (ddd, J = 8.5, 8.5, 0.8Hz, 1H), 2.82 (ddd, J = 8.5, 6.9, 1.2 Hz, 1H), 1.09 (d, J = 6.2 Hz, 3H,). ^{13}C{^{1}H} NMR (DMSO-d_6, 101 MHz): δ (ppm) 163.7, 48.0, 47.9, 21.7.

(3a*R,7a*S**)-Octatrahydro-2-benzimidazolinone (3c)**[5]

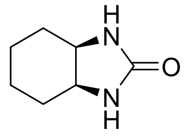

This compound was synthesized, using 1.27 g (8.00 mmol) of **1c**, 76.7 mg (160 μmol, 2 mol%) of $Cp_2Ti(OTf)_2$ and 4.0 mL of DMI and obtained as a white solid according to the typical method.

Yield: 921 mg (6.57 mmol), 82%. ^{1}H NMR (DMSO-d_6, 400 MHz): δ (ppm) 6.12 (br, s, 2H), 3.48-3.38 (m, 2H), 1.65-

1.51 (m, 2H), 1.50-1.35 (m, 4H), 1.30-1.15 (m, 2H). $^{13}C\{^1H\}$ NMR (DMSO-d_6, 101 MHz): δ (ppm) 163.9, 51.0, 28.2, 20.3.

(3a*R,7a*R**)-Octahydro-2-benzimidazolinone (3d)**[5]

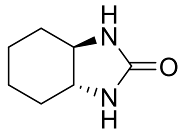

This compound was synthesized, using 316 mg (2.00 mmol) of **1d**, 95.8 mg (200 μmol, 10 mol%) of $Cp_2Ti(OTf)_2$ and 4.5 mL of DMI and obtained as a white solid according to the typical method.

Yield: 173 mg (1.24 mmol), 62%. ^1H NMR (DMSO-d_6, 400 MHz): δ (ppm) 6.31 (br, s, 2H), 2.93-2.78 (m, 2H), 1.95-1.75 (m, 2H), 1.75-1.58 (m, 2H) 1.40-1.20 (m, 4H). $^{13}C\{^1H\}$ NMR (DMSO-d_6, 101 MHz): δ (ppm) 164.8, 60.2, 29.4, 23.7.

3,4,5,6-Tetrahydro-2-pyrimidone (3e)[3]

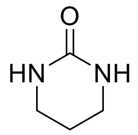

This compound was synthesized, using 1.18 g (10.0 mmol) of **1e**, 95.8 mg (200 μmol, 2 mol%) of $Cp_2Ti(OTf)_2$ and 4.5 mL of DMI and obtained as a pale brown solid. Purification of this compound was carried out by reprecipitation, which was conducted by adding 20 mL of DMSO and 50 mL of Et_2O to the reaction solution, due to low solubility for various solvents.

Yield: 787 mg (7.86 mmol), 79%. ^1H NMR (DMSO-d_6, 400 MHz): δ (ppm) 6.04 (br, s, 2H), 3.07 (dt, J = 2.4 Hz, J = 5.8 Hz, 4H), 1.67 (q, J = 5.8 Hz, 2H). $^{13}C\{^1H\}$ NMR (DMSO-d_6, 101 MHz): δ (ppm) 163.3, 47.5, 47.4, 21.2.

1,3-Dimethyle-2-imidazolidinone (3f)[6]

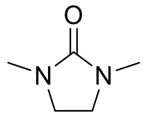

This compound was synthesized at 170 °C for 40 h, using 1.32 g (10.0 mmol) of **1f**, 95.8 mg (200 μmol, 2 mol%) of $Cp_2Ti(OTf)_2$ and 4.5 mL of 1,4-dioxane. After separation by Kugelrohr, this compound was obtained as pale yellow liquid.

Yield: 734 mg (6.43 mmol), 64%. ^1H NMR (DMSO-d_6, 400 MHz): δ (ppm) 3.20 (s, 4H), 2.63 (s, 6H) ppm. $^{13}C\{^1H\}$ NMR (DMSO-d_6, 101 MHz): δ (ppm) 161.3, 44.5, 31.1 ppm.

1,3-Dibenzyle-2-imidazolidinone (3g)[7]

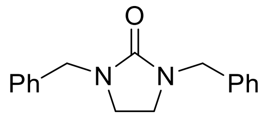

Synthesis of this compound was conducted at 170 °C for 15 h, using 569 mg (2.00 mmol) of **1g**, 95.8mg (200 μmol, 10 mol%) of $Cp_2Ti(OTf)_2$ and 4.5 mL of DMI. After removing the solvent under reduced pressure, this compound was obtained as a colorless oil by column chromatography using diol modified silica with CH_2Cl_2 as an eluent.
Yield: 347 mg (1.30 mmol), 65%. ^{1}H NMR (DMSO-d_6, 400 MHz): δ (ppm) 7.39-7.22 (m, 10H), 4.32 (s, 4H), 3.13 (s, 4H). ^{13}C{^{1}H} NMR (DMSO-d_6, 101 MHz): δ (ppm) 160.4, 137.5, 128.5, 127.7, 127.1, 47.4, 41.7.

3,4-Dihidro-2-quinazolinone (3h)[8]

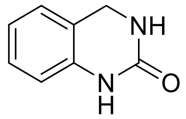

This compound was synthesized, using 1.32 g (4.61 mmol) of **2d**, 76.7 mg (160 μmol, 3.5 mol%) of $Cp_2Ti(OTf)_2$ and 4.0 mL of DMI and obtained as a white solid according to the typical method.
Yield: 587g (3.96 mmol), 85%. ^{1}H NMR (DMSO-d_6, 400 MHz): δ (ppm) 8.99 (br, s, 1H), 7.13-7.03 (m, 2H), 6.87-6.73 (m, 3H), 4.29 (s, 2H). ^{13}C{^{1}H} NMR (DMSO-d_6, 101 MHz): δ (ppm) 154.6, 138.1, 127.6, 125.7, 120.9, 118.1, 113.5, 42.5.

1,3-Dibenzylurea (4a)[5]

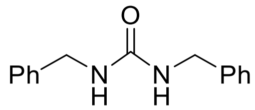

Synthesis of this compound was conducted at 170 °C for 48 h, using 516 mg (2.00 mmol) of **2a**, 95.8 mg (200 μmol, 10 mol%) of $Cp_2Ti(OTf)_2$ and 4.5 mL of DMI and obtained as a white solid according to the typical method.
Yield: 233 mg (0.97 mmol), 48%. ^{1}H NMR (DMSO-d_6, 400 MHz): δ (ppm) 7.35-7.19 (m, 10H), 6.44 (t, J = 6.0 Hz, 2H), 3.13 (d, J = 6.0 Hz, 4H) ppm. ^{13}C{^{1}H} NMR (DMSO-d_6, 101 MHz): δ(ppm) 158.0, 140.8, 128.1, 126.9, 126.4, 42.9.

1,3-Di-*n*-hexylurea (4b)[5]

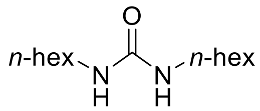

This compound was synthesized at 170 °C for 48 h, using 493 mg (2.00 mmol) of **2b**, 95.8 mg (200 μmol, 10 mol%) of $Cp_2Ti(OTf)_2$ and 4.5 mL of DMI and obtained as a white solid according to the typical method.
Yield: 236 mg (1.03 mmol) 52%. ^{1}H NMR (DMSO-d_6, 400 MHz): δ (ppm) 5.70 (t, J = 5.6 Hz, 2H), 2.94 (dt, J = 6.7, 5.6 Hz, 4H), 1.40-1.18 (m, 16H), 0.86 (t, J = 6.8 Hz, 6H). ^{13}C{^{1}H} NMR (DMSO-d_6, 101 MHz): δ (ppm) 157.9, 39.1, 30.9, 29.9,25.9, 22.0, 13.8.

1,3-Dicyclohexylurea (4c)[5]

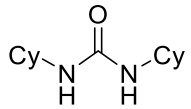

This compound was synthesized at 170 °C for 15 h, using 484 mg (2.00 mmol) of **2c**, 95.8 mg (200 μmol, 10 mol%) of $Cp_2Ti(OTf)_2$ and 4.5 mL of DMI. Yield of this compound was obtained as NMR yield in DMSO-d_6 containing 1,3,5-trimethylebenzene as an internal standard.
Yield: 13%.

Copies of NMR spectra for the synthesized urea derivatives

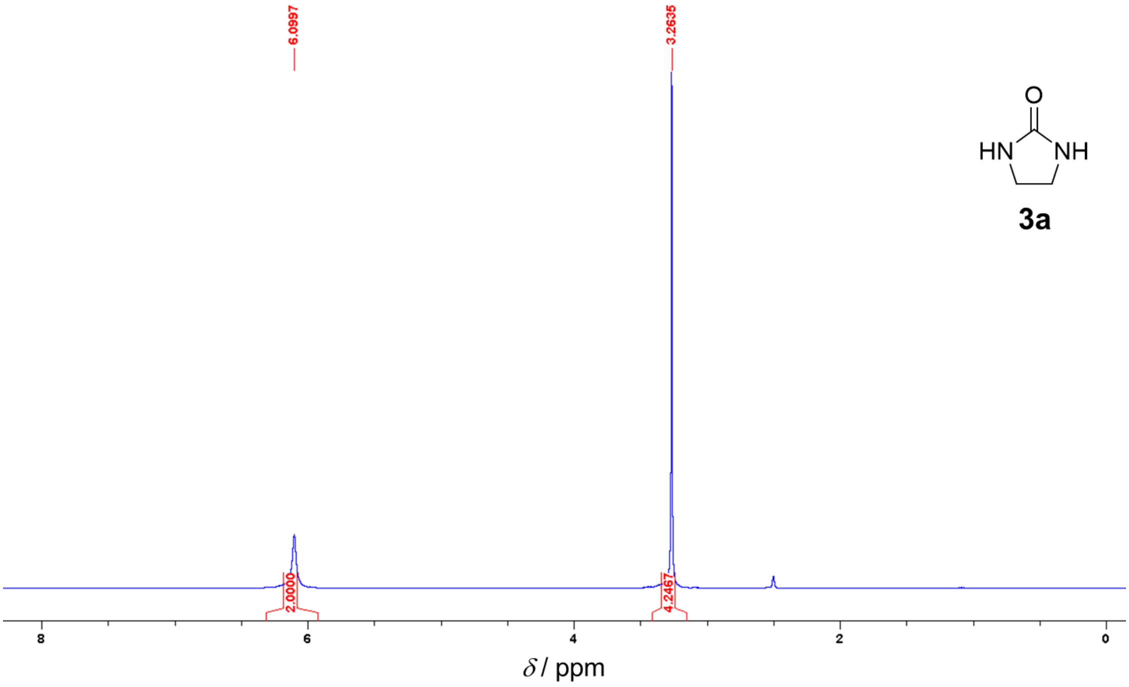

Supplementary Fig. 6| ^{1}H NMR spectrum of **3a**.

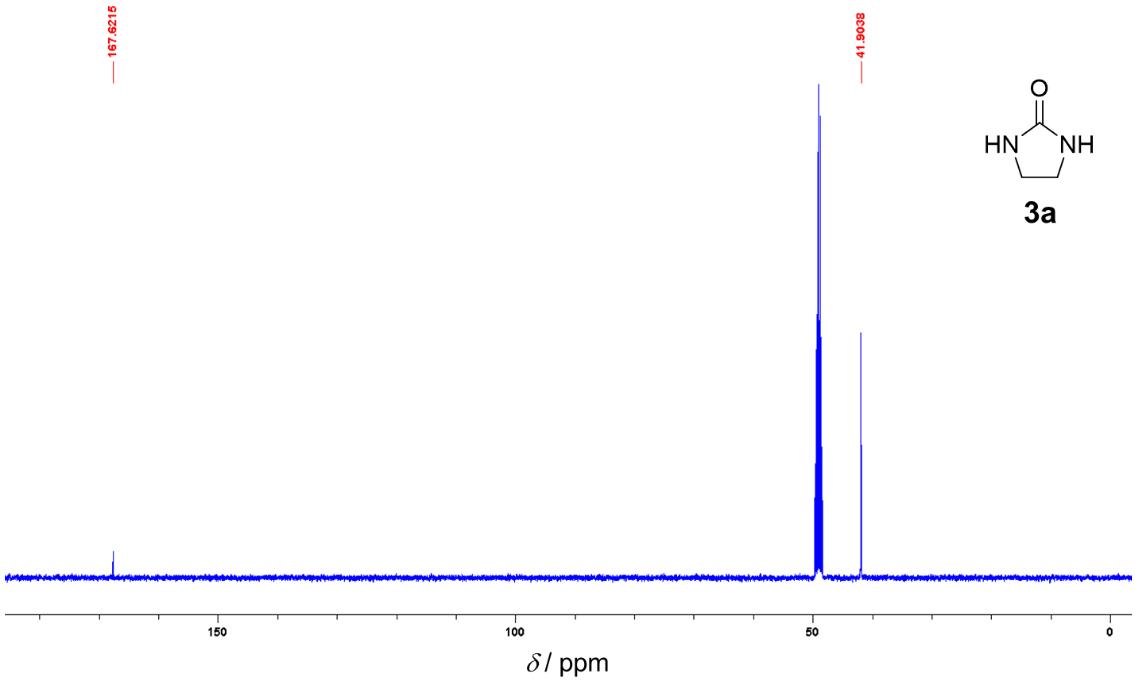

Supplementary Fig. 7| $^{13}C\{^{1}H\}$ NMR spectrum of **3a**.

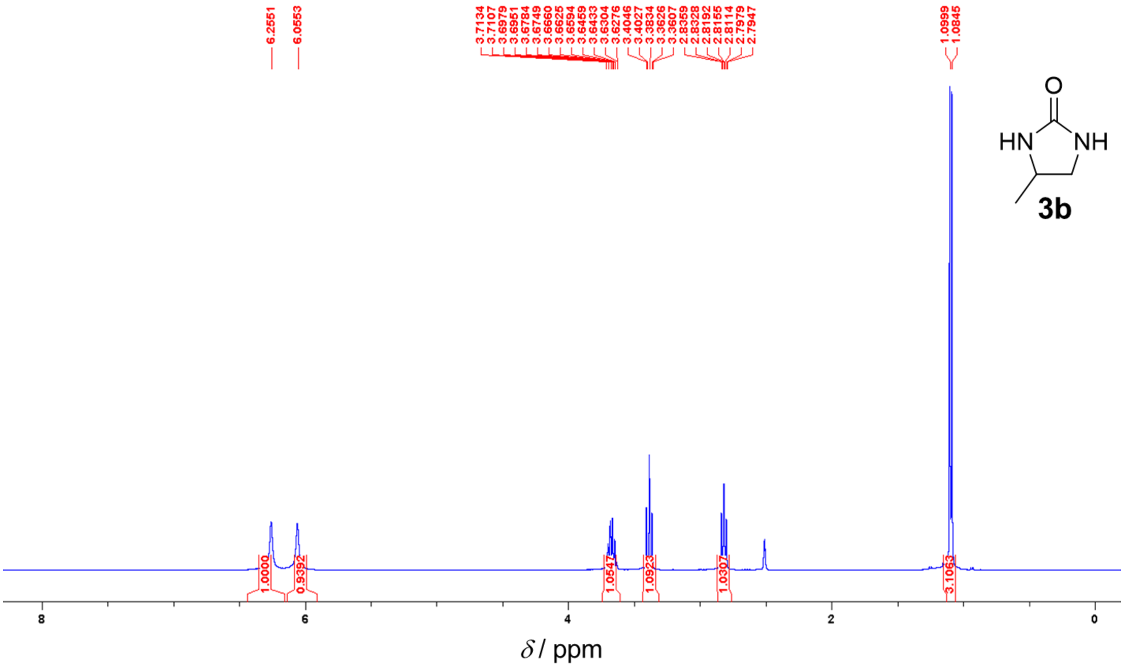

Supplementary Fig. 8| ^{1}H NMR spectrum of **3b**.

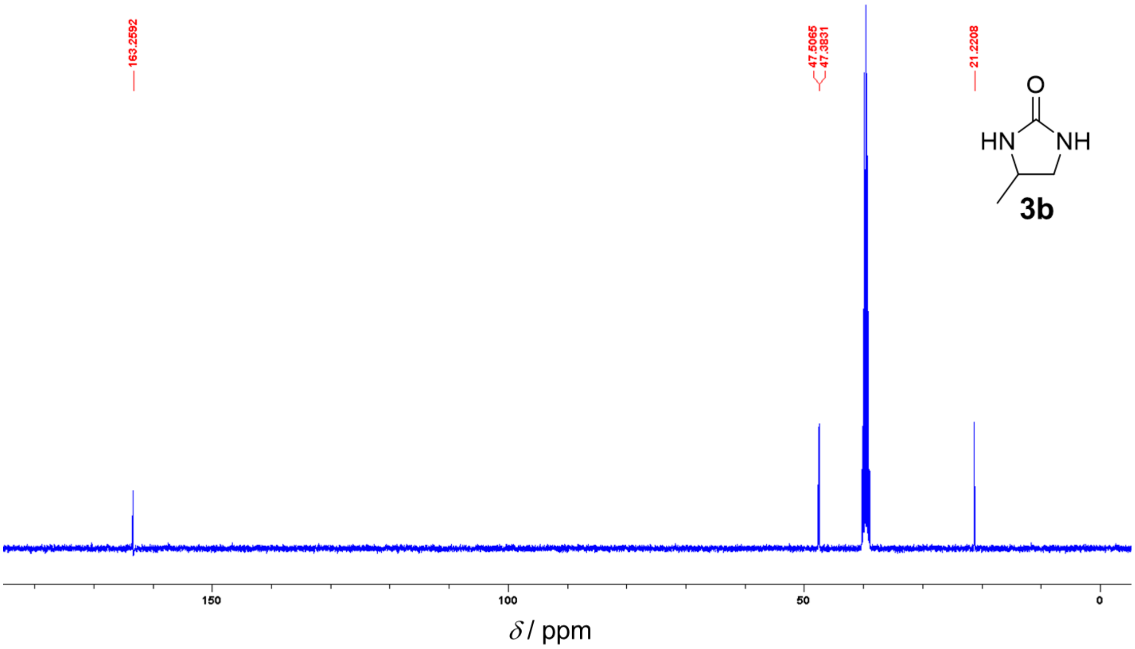

Supplementary Fig. 9| ^{13}C{^{1}H} NMR spectrum of **3b**.

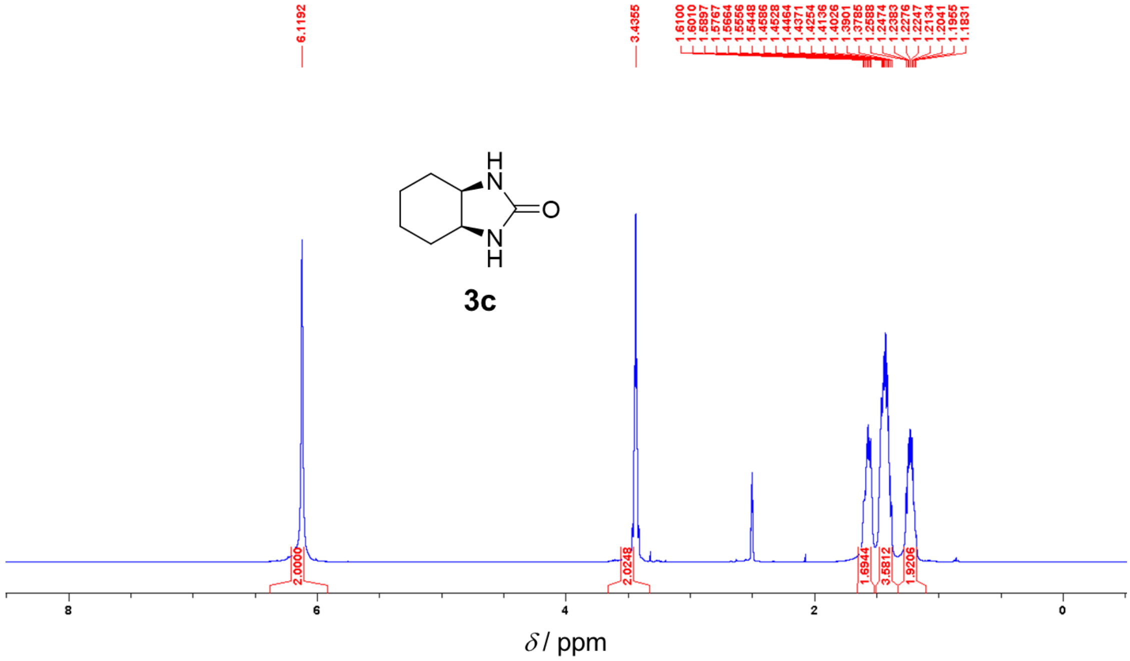

Supplementary Fig. 10| ^{1}H NMR spectrum of **3c**.

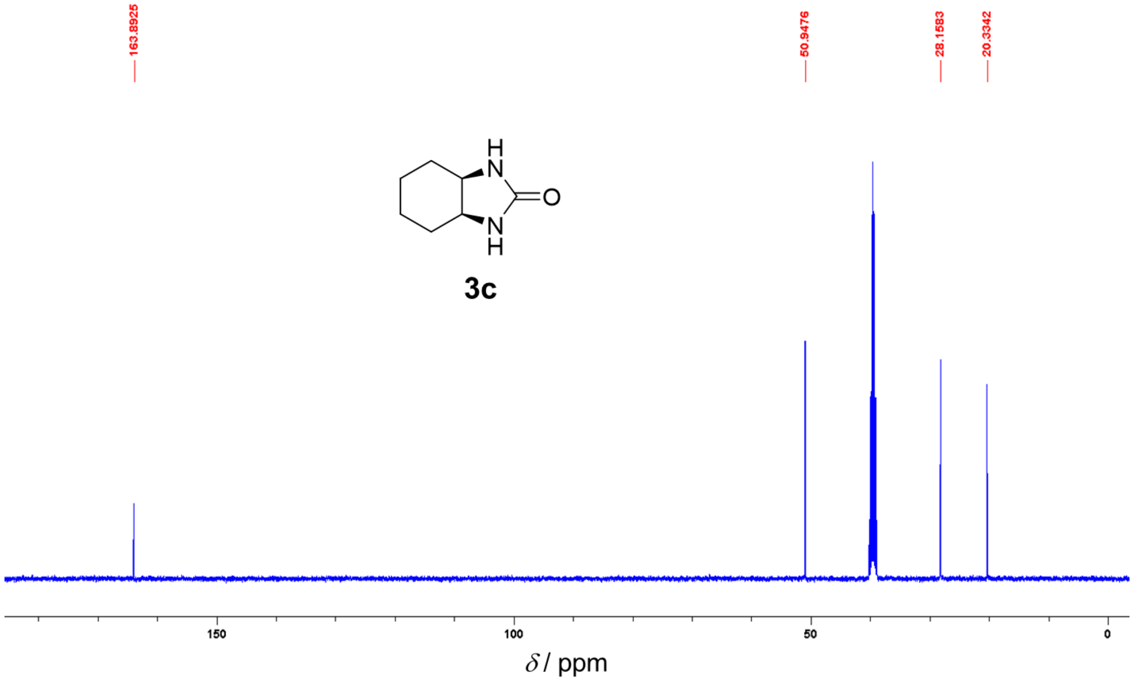

Supplementary Fig. 11| ^{13}C{^{1}H} NMR spectrum of **3c**.

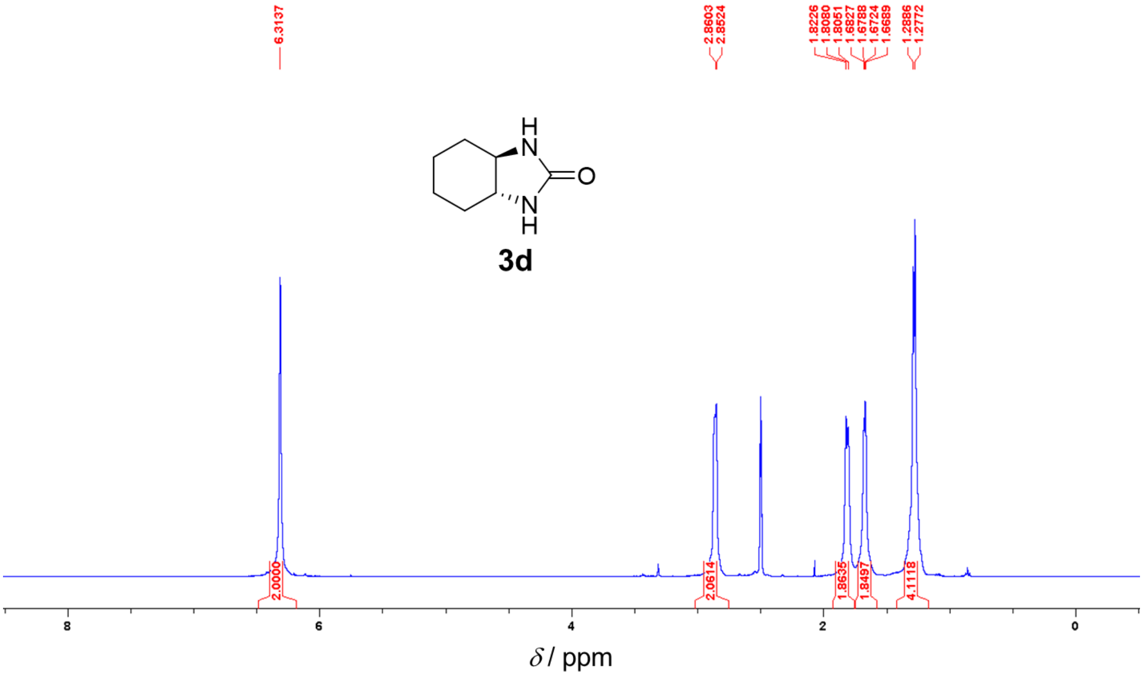

Supplementary Fig. 12| ^{1}H NMR spectrum of **3d**.

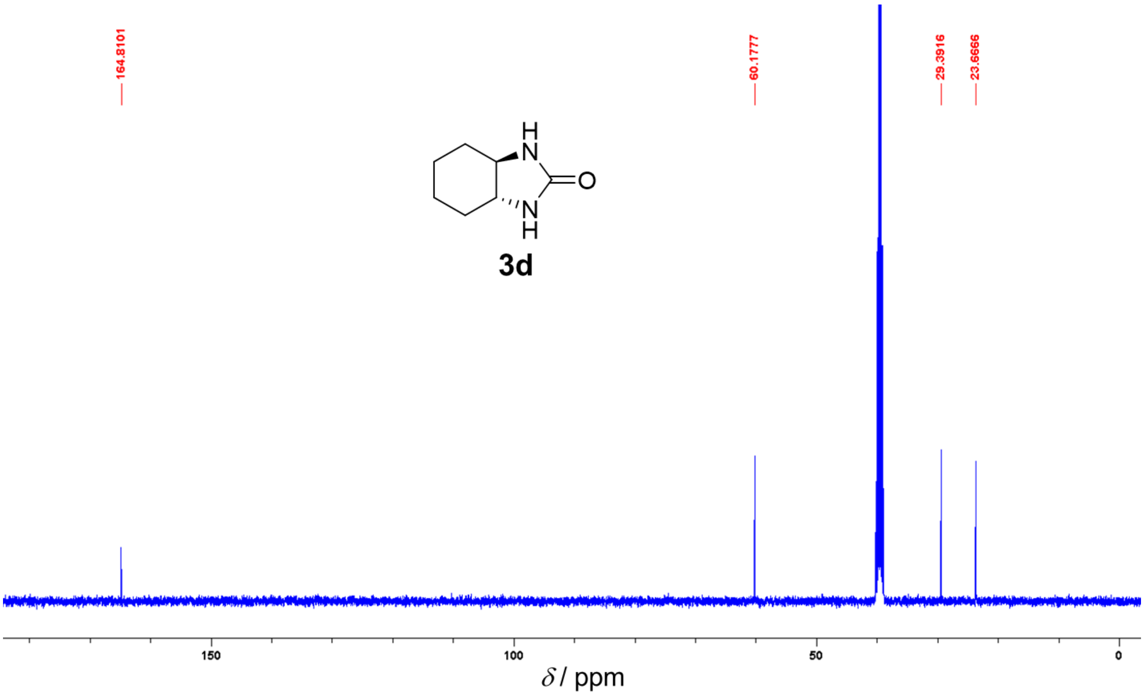

Supplementary Fig. 13| $^{13}C\{^{1}H\}$ NMR spectrum of **3d**.

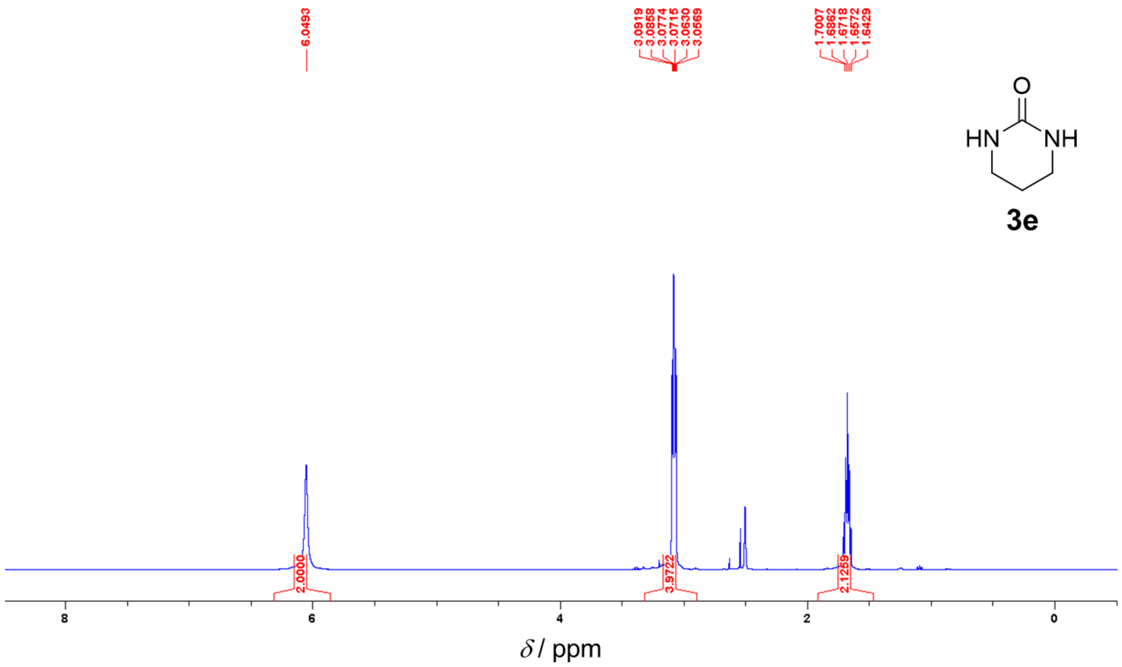

Supplementary Fig. 14| ^{1}H NMR spectrum of **3e**.

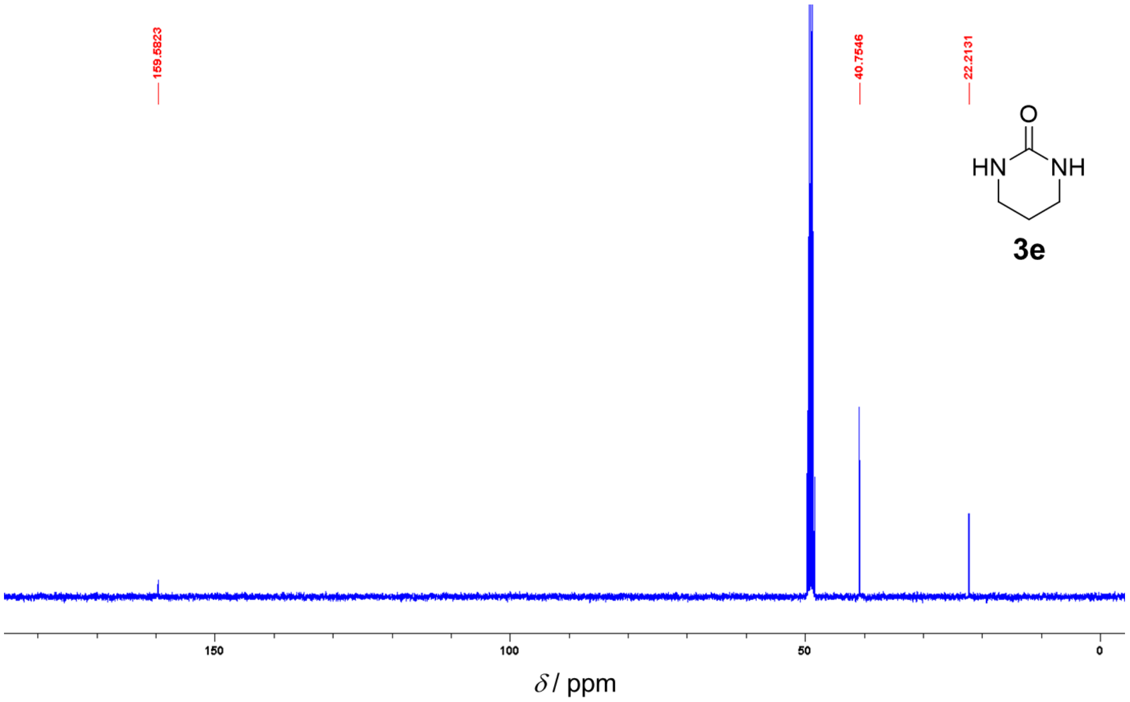

Supplementary Fig. 15| $^{13}C\{^{1}H\}$ NMR spectrum of **3e**.

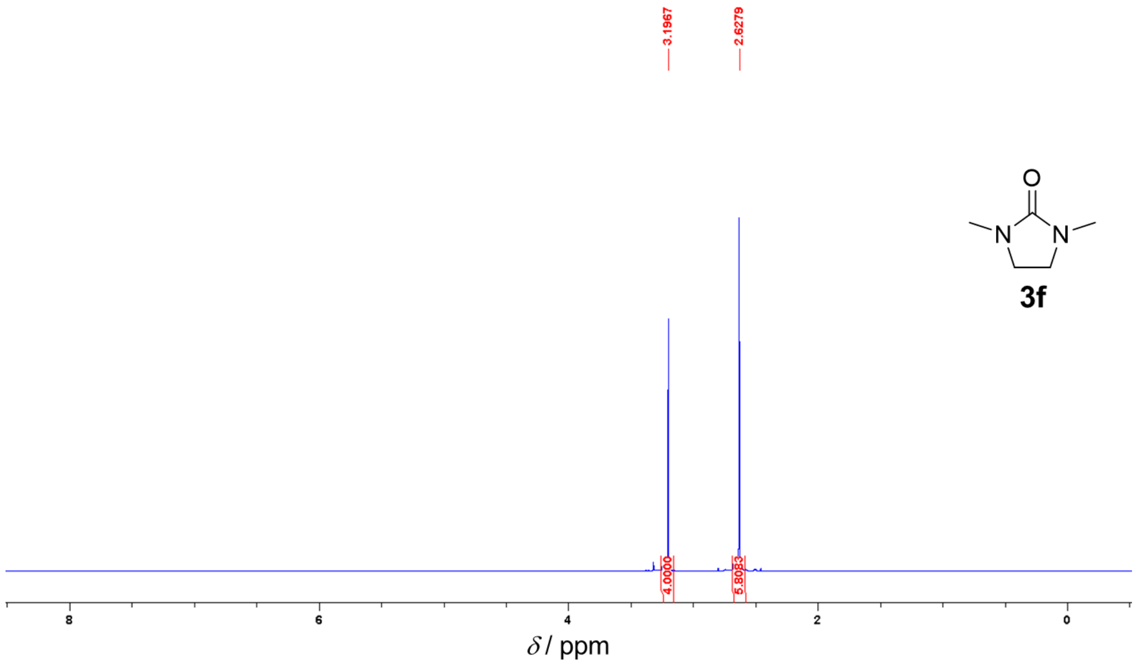

Supplementary Fig. 16| ^{1}H NMR spectrum of **3f**.

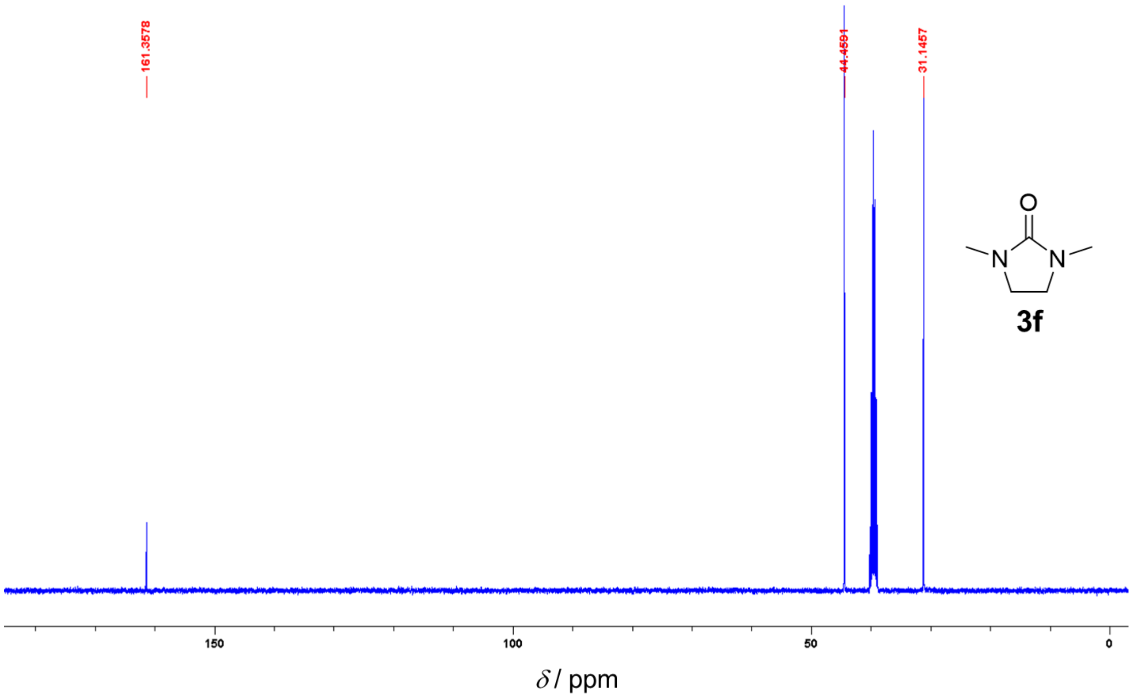

Supplementary Fig. 17| $^{13}C\{^{1}H\}$ NMR spectrum of **3f**.

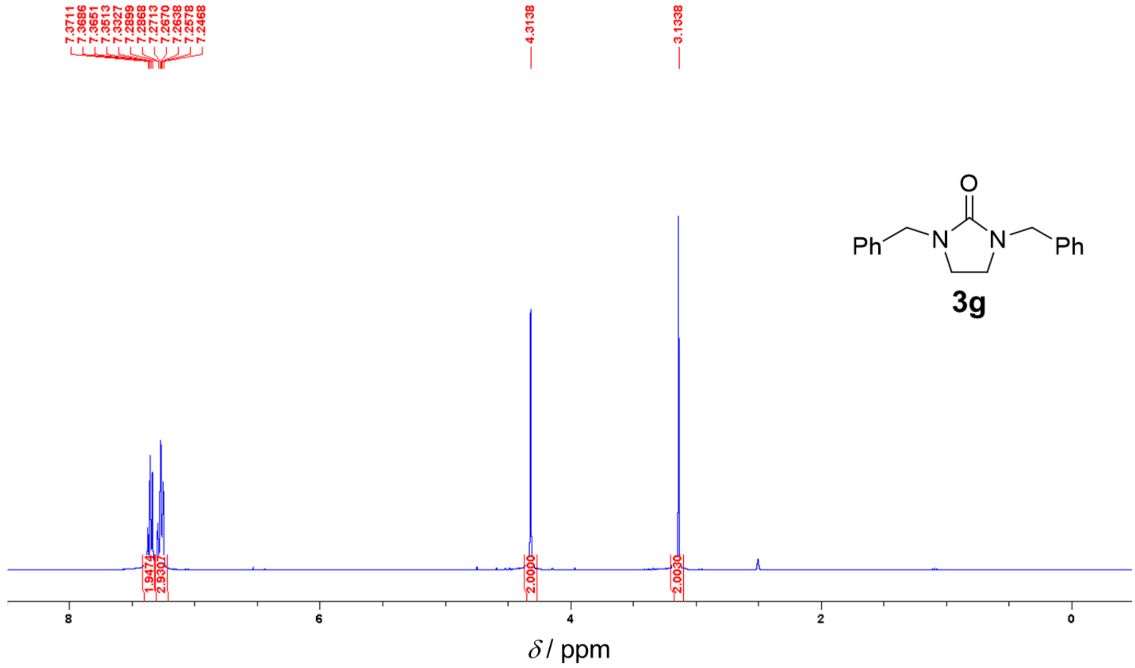

Supplementary Fig. 18| ^{1}H NMR spectrum of **3g**.

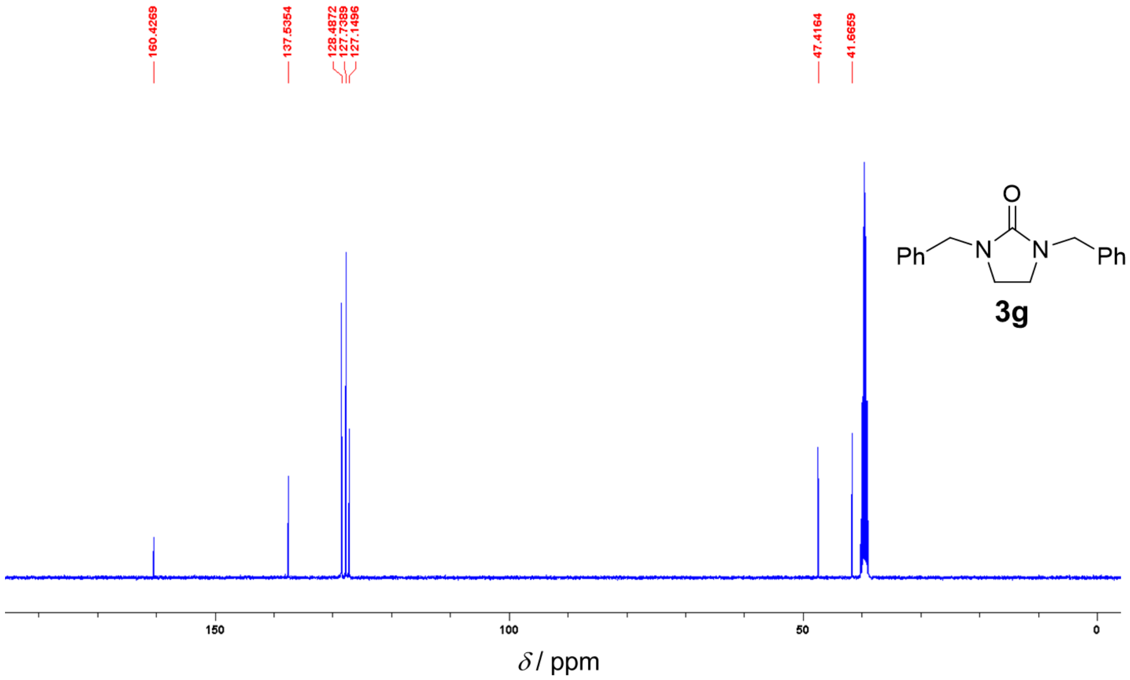

Supplementary Fig. 19| ^{13}C{^{1}H} NMR spectrum of **3g**.

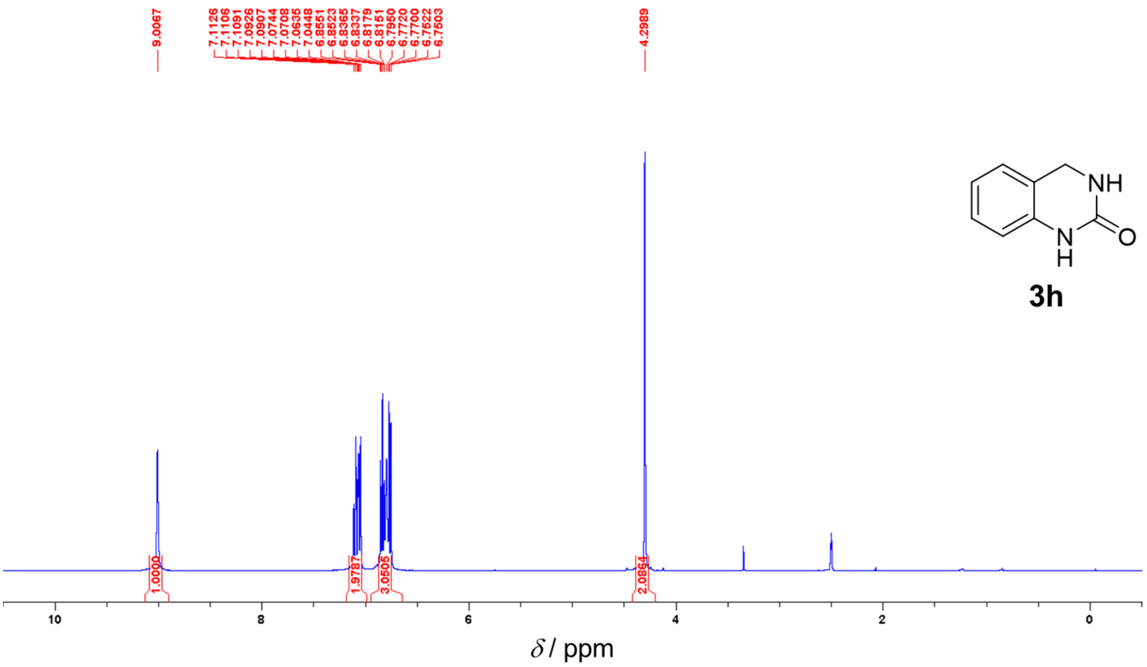

Supplementary Fig. 20| ^{1}H NMR spectrum of **3h**

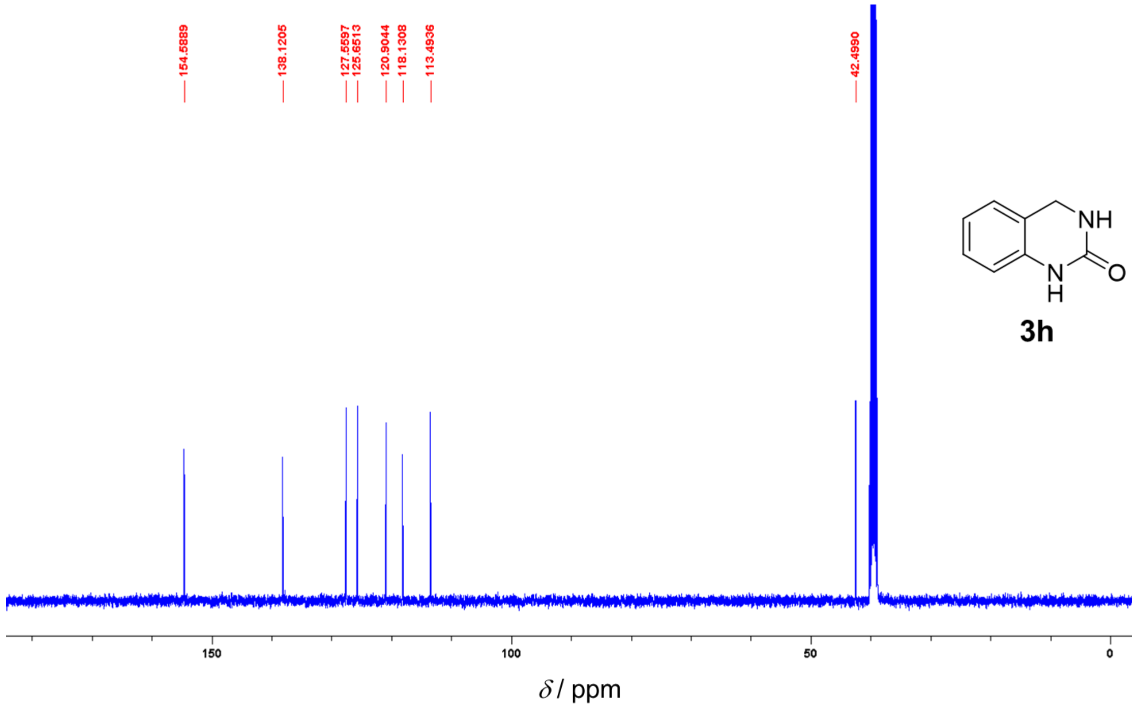

Supplementary Fig. 21| ^{13}C NMR spectrum of **3h**

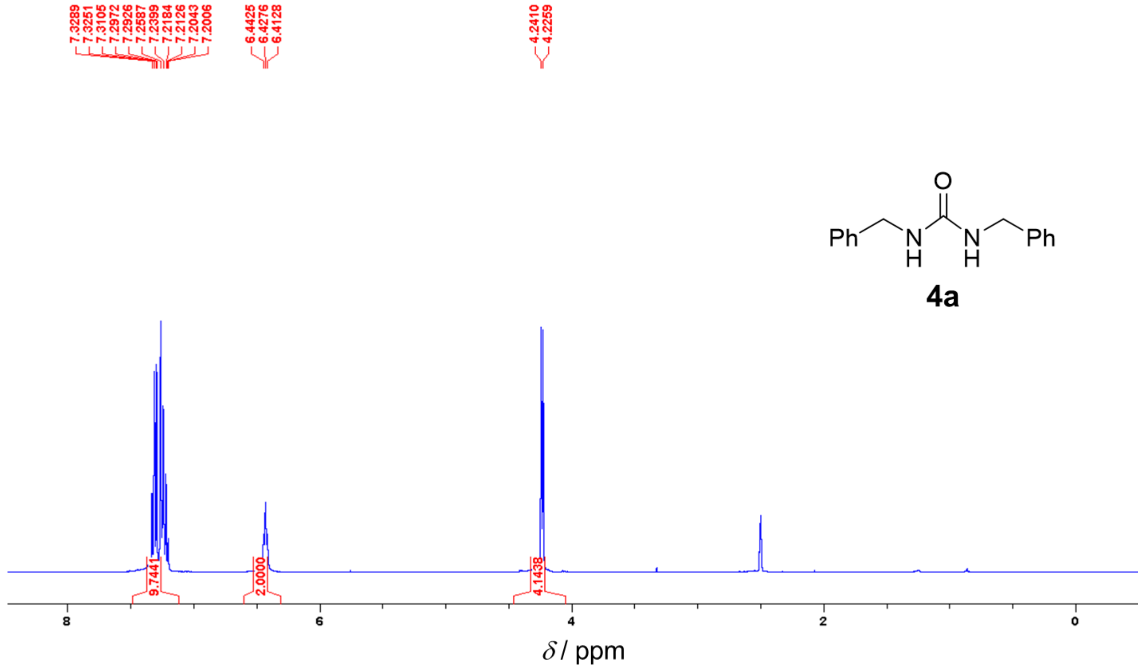

Supplementary Fig. 22| ^{1}H NMR spectrum of **4a**.

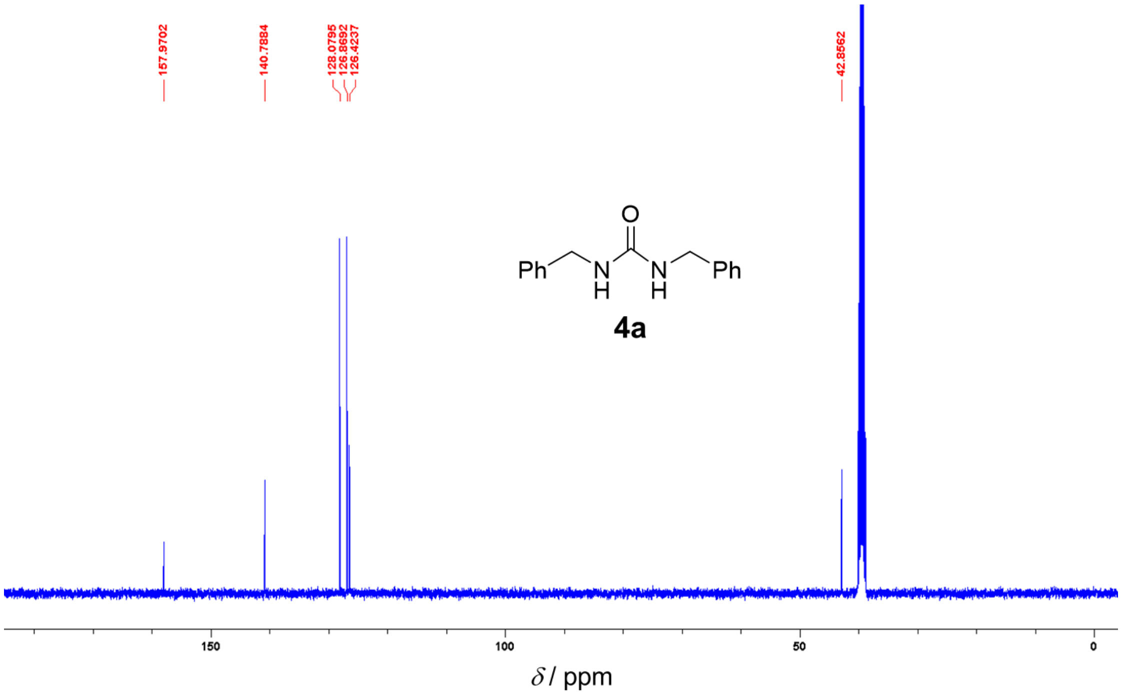

Supplementary Fig. 23| ^{13}C{^{1}H} NMR spectrum of **4a**.

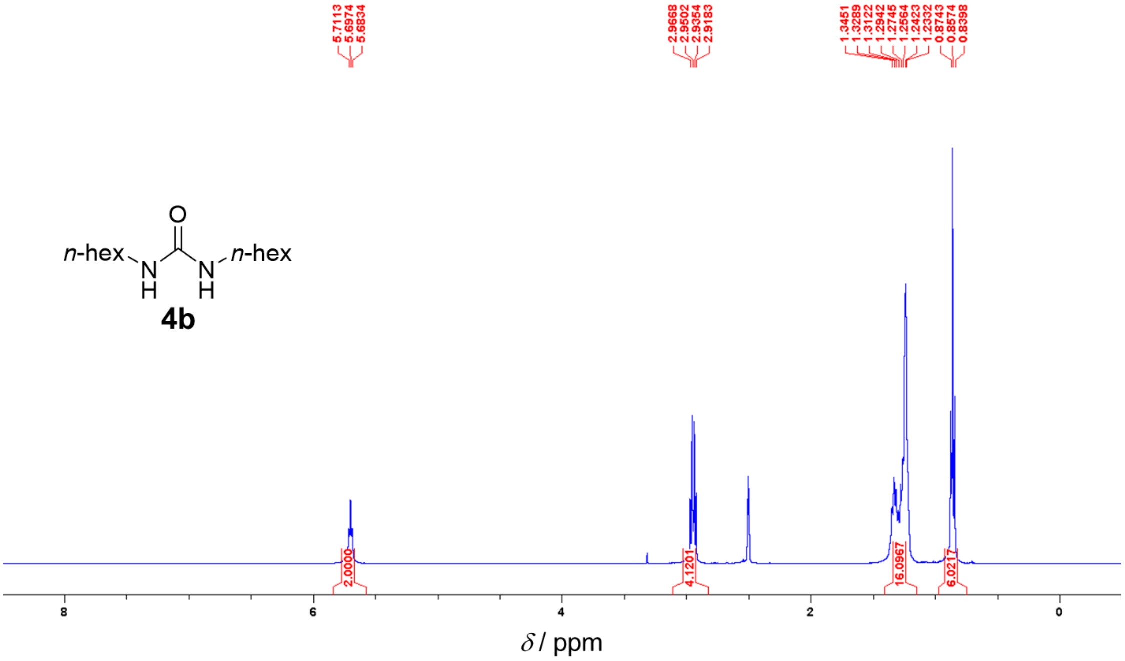

Supplementary Fig. 24| ^{1}H NMR spectrum of **4b**.

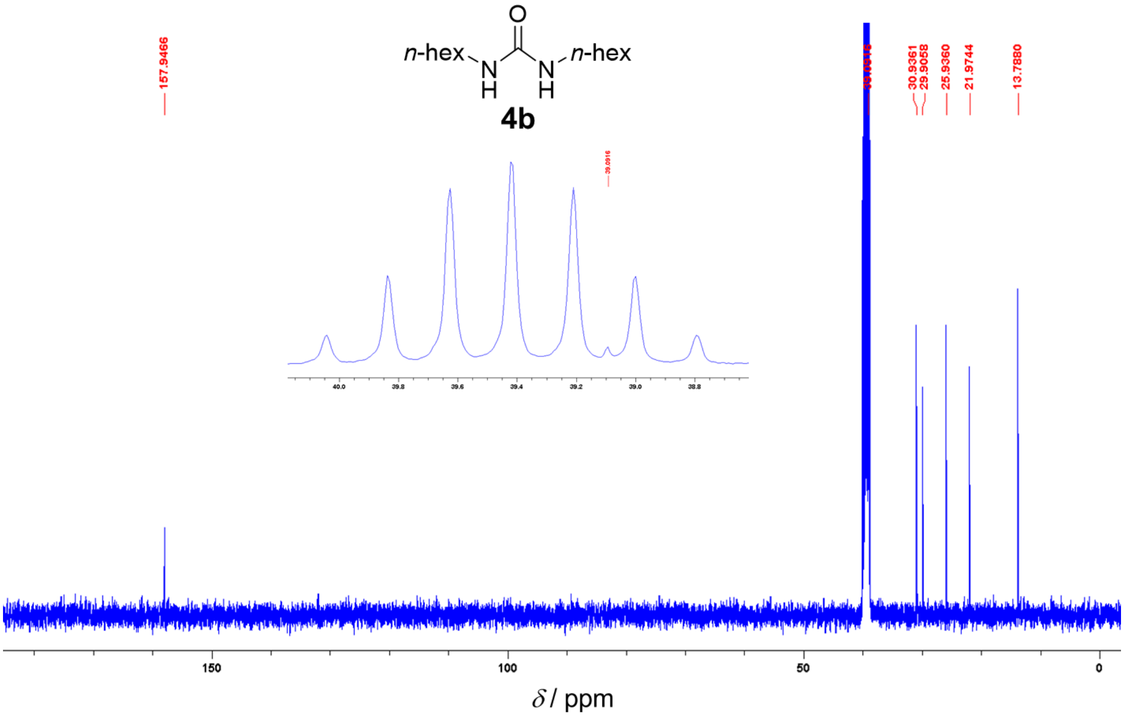

Supplementary Fig. 25| ^{13}C{^{1}H} NMR spectrum of **4b**.

One-pot synthesis of urea derivatives

2 mmol of diamine was mixed into 2 mL of DMI in a 5 mL autoclave reactor vessel and shielded with a ring gasket and a lid with two-way cock. CO_2/N_2 mixed gas (v:v = 15:85) was vented into only gas phase through the branch using syringe needles and a septum at constant flow rate (20 mL/min) controlled by a mass flow controller for 60 min to prevent clogging of a needle tip by precipitated ammonium carbamate derivatives. Then, this gas was bubbled for the reaction mixture for 30 min with the same flow rate. After that, the lid and the ring gasket were removed from the vessel and 95.2 mg of $Cp_2Ti(OTf)_2$ (0.2 mmol, 10 mol%) solved in 2.5 mL of DMI was added to the vessel in a N_2 filled glove box. This vessel was completely shielded with a stainless plate gasket and dunked in a heated oil bath. the reaction solution was stirred at 170 ºC for 24 h. After reaction, the vessel was removed from the oil bath and cooled to room temperature. The reaction mixture was extracted with MeOH (ca. 20 mL) and 1,3,5-trimethylebenzene (~100 mg) was added as an internal standard for the ^{1}H NMR. The ^{1}H NMR spectra were measure in DMSO-d_6.

Pressure change during reaction of 3a synthesis

A reaction of **3a** synthesis to measure inner pressure change was conducted in 5 mL autoclave reactor vessel with a ring type gasket and a lid with pressure gage at 170 ºC for 24 h, using 95.2 mg of $Cp_2Ti(OTf)_2$ (0.2 mmol, 2 mol%), 1041 mg of **1a** (10 mmol) and 4.5 mL of DMI. The preparation was conducted in a N_2 filled glove box.

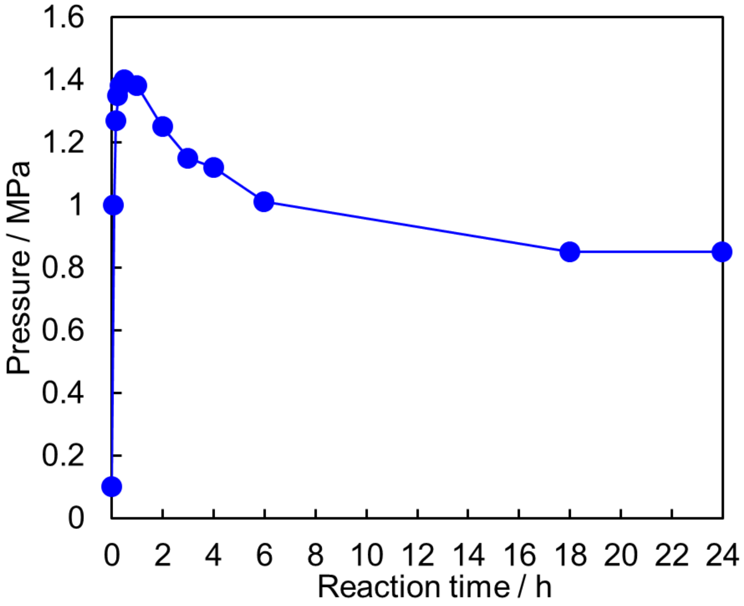

Supplementary Fig 26| Time course of inner pressure during **3a** synthesis.

Supplementary References

1. Luinstra, G. A. Synthesis and reactivity of titanocene and zirconocene triflates. *J. Organomet. Chem.* **517**, 209–215 (1996).
2. Fulmer, G. R. *et al.* NMR Chemical Shifts of Trace Impurities: Common Laboratory Solvents, Organics, and Gases in Deuterated Solvents Relevant to the Organometallic Chemist. *Organometallics* **29**, 2176–2179 (2010).
3. Petzold, D. *et al.* Visible-Light-Mediated Liberation and In Situ Conversion of Fluorophosgene. *Chem. Eur. J.* **25**, 361–366 (2019).
4. Mizuno, T., Takahashi, J. & Ogawa, A. Synthesis of 2-oxazolidones by sulfur-assisted thiocarboxylation with carbon monoxide and oxidative cyclization with molecular oxygen under mild conditions. *Tetrahedron* **58**, 7805–7808 (2002).
5. Smirnova, E. S. *et al.* Polynuclear Gold $[Au^{I}]_4$, $[Au^{I}]_8$, and Bimetallic $[Au^{I}_4Ag^{I}]$ Complexes: C−H Functionalization of Carbonyl Compounds and Homogeneous Carbonylation of Amines. *Angew. Chem. Int. Ed.* **55**, 7487–7491 (2016).
6. Mizuno, T., Nakai, T. & Mihara, M. Novel synthesis of *N,N′*-dialkyl cyclic ureas using sulfur-assisted carbonylation and oxidation. *Heteroat. Chem.* **20**, 64–68 (2009).
7. Semba, K., Ohta, N. & Nakao, Y. Carboallylation of Electron-Deficient Alkenes with Organoboron Compounds and Allylic Carbonates by Cooperative Palladium/Copper Catalysis. *Org. Lett* **21**, 4407–4410 (2019).
8. Xu, M. *et al.* Synthesis of Urea Derivatives from CO_2 and Silylamines. *Angew. Chem. Int. Ed.* **58**, 5707–5711 (2019).
